# Monitoring and Evaluating Progress towards Universal Health Coverage in Bangladesh

**DOI:** 10.1371/journal.pmed.1001722

**Published:** 2014-09-22

**Authors:** Tanvir Huda, Jahangir A. M. Khan, Karar Zunaid Ahsan, Kanta Jamil, Shams El Arifeen

**Affiliations:** 1 International Centre for Diarrhoeal Disease Research, Bangladesh (ICDDR,B), Dhaka, Bangladesh; 2 School of Public Health, Sydney Medical School, University of Sydney, Sydney, Australia; 3 MEASURE Evaluation, University of North Carolina, Chapel Hill, North Carolina, United States of America; 4 United States Agency for International Development/Bangladesh, Dhaka, Bangladesh

## Abstract

This paper is a country case study for the Universal Health Coverage Collection, organized by WHO. Tanvir Mahmudul Huda and colleagues illustrate progress towards UHC and its monitoring and evaluation in Bangladesh.

*Please see later in the article for the Editors' Summary*

This paper is part of the PLOS Universal Health Coverage Collection. This is the summary of the Bangladesh country case study. The full paper is available as Supporting Information file Text S1.

## Background

In the 42 years since independence, Bangladesh has made some substantial progress in the health sector, which is all the more remarkable when compared with other countries in the region [1]–[5]. However, this achievement is not uniform across all health indicators. The coverage of many critical health services is still quite low. The country's health system is struggling to meet basic standards for quality of care because of a shortage of skilled health workers, the large number of unregulated private service providers, irregular supplies of drugs, inadequate public financing, high out-of-pocket expenses, and lack of proper monitoring and supervision mechanisms. Further complicating the situation is the increasing burden of non-communicable diseases, and the absence of any pre-payment risk pooling mechanisms. Bangladesh faces a daunting challenge in achieving the goal of universal health coverage (UHC).

## Universal Health Coverage: The Policy Context

In recent years, there has been a general increase in media coverage about UHC and efforts to learn from neighboring countries, which has contributed to a policy-level dialogue on UHC in Bangladesh. The first ever health financing strategy for the country was developed and approved in 2012 with a roadmap to achieve UHC by 2032 [6]. Despite this apparent momentum, there has been remarkably little implementation of any UHC initiatives on the ground [7].

## Monitoring and Evaluation of Universal Health Coverage

### Results Framework for the National Health Sector Program and Source of Data

The country has established an annual process to assess the progress of its Health, Population and Nutrition Sector Development Programme (HPNSDP) on the basis of a results framework [8]. Our assessment of the results framework reveals that there is a gap in monitoring critical program inputs and processes required for the management of health system investments. Indicators measuring service access for non-communicable diseases and injuries are missing at the output level. Further, there is no indicator related to the measurement of coverage of secondary or tertiary level health care services, or risk factors for non-communicable diseases at the outcome level. Most importantly, indicators that measure coverage of financial risk protection—a key component of UHC—are completely absent.

### Monitoring and Evaluation Framework for Universal Health Coverage

Bangladesh needs to assess progress through a well-defined monitoring and evaluation framework to move towards UHC. The country needs to continue investing substantially in strengthening the capacity of its weak health system in order to make UHC a reality. Thus, using a set of indicators that are comparable with other countries, it would be critical to track health financing, the density of the health workforce, supplies of essential medicines, the availability and functionality of key instruments, and the use of the health information system. The government should continue to measure coverage of priority public-health interventions and include those for non-communicable diseases for all ages and gender. The country will also need continuous feedback about whether efforts towards achieving UHC are also contributing to progressive realization of the equity goals [8].

## Progress towards UHC in Bangladesh

**Figure 1:**
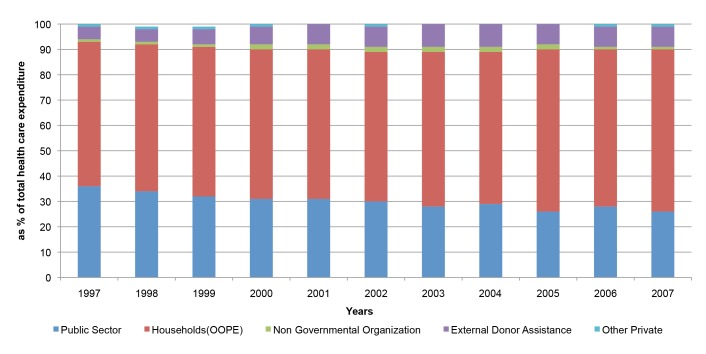
Trends in health care expenditure 1997–2007 [9].

At the input level, health-financing data show that Bangladesh's total health care expenditure steadily increased from 1997 to 2007. The growth in total health expenditures, however, is mostly because of the accelerated growth in out-of-pocket expenditures. In 1997, household health expenditure constituted 57% of total health expenditure, which increased to 64% in 2007 (Figure 1) [9].

**Figure 2:**
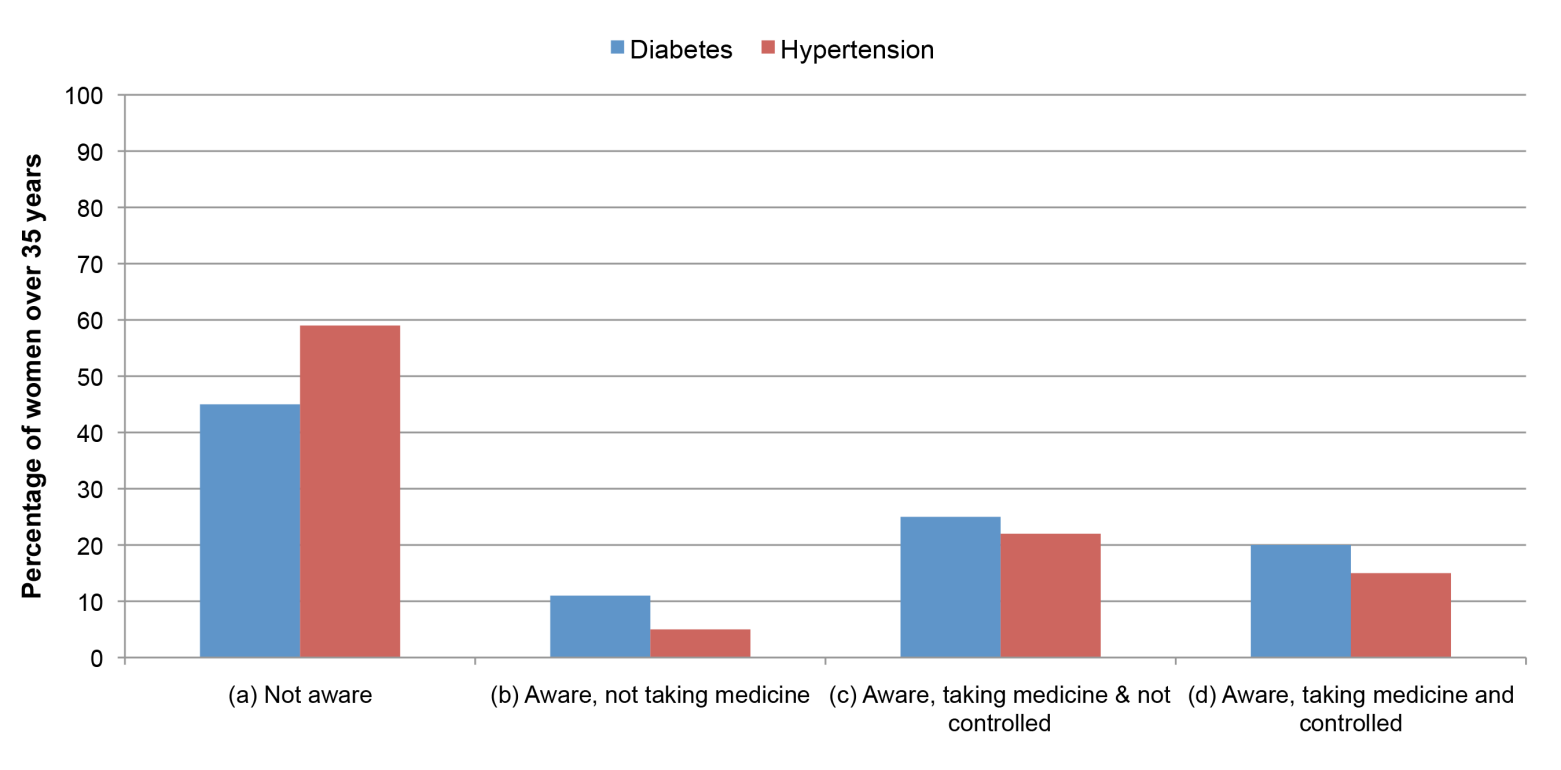
Hypertension and diabetes awareness and treatment status in women age 35 and over. (a) The percent of women who have hypertension (defined as blood pressure values of systolic blood pressure greater than or equal to 140 mmHg or diastolic blood pressure greater than or equal to 90 mmHg) and diabetes (defined as fasting plasma glucose levels greater than or equal to 7.0 mmol/l) but not aware of the condition. (b) The percent of women who are aware of their condition but not taking medicine. (c) The percent of women who are aware of their condition, taking medication, but the condition is not under control. (d) The percent of women who are aware of their condition, taking medication, and the condition is under control (receiving effective treatment) [10].

In terms of population coverage, the country has reached fairly high levels of coverage with greater equity for several priority public-health interventions. In 2011, 86% of children ages 12–23 months were immunized with all basic vaccines, of which 77% were in the lowest wealth quintile and 94% in the highest wealth quintile. Primary treatment coverage for diarrhoea and acute respiratory infections (ARIs) has also improved. In 2011 81% of children under-five years old with diarrhoea were treated with oral rehydration salts. Among children with ARI, 35% were taken to a health facility or a health care provider and 71% received an antibiotic. However, the country has done less well with certain interventions that require relatively higher levels of clinical care, For example, skilled birth attendants assisted in only 32% of all deliveries [10].Bangladesh has a high prevalence of major non-communicable diseases. The Bangladesh demographic and health 2011 survey reported that 32% of women and 19% of men older than 35 years of age are hypertensive. For diabetes these figures are 11.2% and 10.7%, respectively [10]. The same survey reported that 20% of women with hypertension and 15% of women with diabetes had their conditions poorly control with medication (Figure 2). For men these figures are 16% and 10% [10]. At population impact level, Bangladesh is well on its way to achieving the main targets of both Millennium Development Goals 4 and 5, with plummeting rates of under-five and maternal mortality [10]–[13]. However the rate of stunting is still high, although slowly declining.

## Conclusions and Recommendations

Bangladesh faces enormous challenges in achieving UHC. The government needs to invest heavily in health to strengthen the capacity of its weak health system. It also urgently needs to introduce mandatory pre-payment schemes for formal sector employees and subsidized schemes for the poor population as planned in the health financing strategy. In terms of monitoring the progress of UHC, it is important to identify achievable and measurable milestones adapted to its own context. The UHC monitoring and evaluation framework must be established through inclusive policy dialogue and effectively reflect the country's disease burden profile, health system capacity, and level of economic development.

The country needs to strengthen its information sources. Among the major information sources, the routine health information system represents perhaps the most pressing area for improvement, as it is likely the most crucial component of the successful monitoring and evaluation of both the health sector program and UHC. Strengthening the civil vital registration system is almost as urgent as the need to strengthen the routine health information system. It would also be important to strengthen the Ministry of Health and Family Welfare's stewardship capacity so that information from the private sector is collected in the future through the routine health information system. It is expected that the country will make effectively and reliably tracking progress towards achieving UHC the highest priority. A strong appreciation of what is needed to achieve UHC and political commitment from the highest levels in the country are also absolute prerequisites.

## Supporting Information

Text S1 **The full country case study for Bangladesh.**
(DOCX)

## Abbreviations

UHC: universal health coverage
