# Supplementary material for: Monitoring and Evaluating Progress towards Universal Health Coverage in Bangladesh
Source: PLoS Med. 2014 Sep 22;11(9):e1001722. doi: 10.1371/journal.pmed.1001722 (PMC4170958; doi:10.1371/journal.pmed.1001722)
Supplement: Text S1 — The full country case study for Bangladesh. (DOCX) [file pmed.1001722.s001.docx]

**Full Case Study: Monitoring and evaluating progress towards Universal Health Coverage in Bangladesh**

Tanvir Huda^1,2*^, Jahangir A. M. Khan^1^, Karar Zunaid Ahsan^3^, Kanta Jamil^4^ and Shams El Arifeen^1^

^1^ Centre for Child and Adolescent Health, International Centre for Diarrhoeal Disease Research, Bangladesh (ICDDR,B), Dhaka, Bangladesh

^2^ School of Public Health, Sydney Medical School, University of Sydney, Sydney, Australia

^3^ MEASURE Evaluation, University of North Carolina, Chapel Hill, USA

^4^ United States Agency for International Development/Bangladesh, Dhaka, Bangladesh

*Corresponding author: Tanvir Huda

Email: thuda@icddrb.org; huda.tanvir@gmail.com

**This paper is the full country case study to accompany the summary paper “Monitoring and evaluating progress towards Universal Health Coverage in Bangladesh” that is part of the Universal Health Coverage Collection. Not commissioned; externally reviewed.**

**Abstract:** The health sector in Bangladesh has shown significant progress especially in child, maternal health and family planning. However the country is still far behind in providing required health care services to its citizen that are of adequate quality at an affordable price, and thus achieving the goal of Universal Health Coverage (UHC). It urgently needs to develop and implement a comprehensive agenda for UHC with an appropriate monitoring and evaluation (M&E) framework to track the progress towards UHC. The UHC M&E framework should be based on the country’s disease burden profile, health system capacity and level of economic development. The current health sector program’s results framework could be the basis for the UHC M&E framework, however indicators that effectively measure the coverage of all essential interventions for non-communicable diseases, injuries and different dimensions of health financing need to be included. It is also imperative that UHC M&E framework is embedded in the country’s overall strategic plan for the health sector. The country needs to strengthen its routine health information system as well as its civil vital registration system. It also must strengthen the Ministry of Health’s stewardship capacity so that information from the private sector can be made available routinely for more effective tracking of progress towards UHC.

**Summary Points:**

1. Bangladesh faces a daunting challenge in achieving the goals of UHC, because of its underperforming health care system, an absence of effective prepayment schemes, and the double burden of communicable and non-communicable diseases
2. The country must develop and implement a comprehensive agenda for UHC with an appropriate M&E framework covering all levels of health system performance
3. The M&E framework must cover health financing inputs, and coverage of prepayment schemes to provide adequate financial protection to its citizens
4. Bangladesh has done remarkably well in maternal and child health; however coverage of essential services for non-communicable diseases, nutrition, and diseases that require higher levels of intervention is still low
5. For more effective tracking of progress towards UHC, it must strengthen its routine health information system, civil vital registration and government stewardship in ensuring availability of data from the private sector
6. A strong appreciation of what is needed to achieve UHC and political commitment from the highest levels in the country is an absolute prerequisite.

**1. Background**

Universal health coverage (UHC) is defined as a situation where all people who need health services receive them without the financial hardship associated with payment [1]. The concept of UHC was first endorsed by the World Health Assembly in 2005, followed by the unanimous adoption of the UHC resolution by UN General Assembly in 2012[2]. Since then, many countries have adopted the UHC agenda and are considering major health system reform towards that goal. Governments are increasingly recognizing the importance of good health in achieving their development goals, and have implemented initiatives to provide affordable and quality health services to their citizens.

In the 42 years since independence, Bangladesh has made some significant progress in the health sector. The recent Lancet series on Bangladesh has affirmed the country’s exceptional achievements in a number of areas of health, which are all the more remarkable when compared with other countries in the region [3-7]. However, this achievement is not uniform across all health indicators. One particular example is in child and maternal health. Despite steep reductions in the mortality rates and staying on track to achieve the MDG 4 and 5 goals, the coverage of several critical child and maternal health services is still quite low, and is compounded by persistent high prevalence of malnutrition among both mothers and children[3, 8-10]. With the limited data available, it appears that Bangladesh doing poorly in providing services directed towards non-communicable diseases, injuries, diseases that require clinical diagnosis, surgical interventions and conditions mostly suffered by the elderly.

In Bangladesh the Ministry of Health and Family Welfare (MOHFW) is the major health care service provider for all kinds of health services. Currently the public health delivery infrastructure consists of about 12,000 community clinics, approximately 7,000 health and family welfare centres including Maternal, Neonatal and Child Health and Family Planning (MNCH&FP) clinics, 459 upazilla (sub-district) hospitals, and 124 secondary and tertiary hospitals[4]. National and International NGOs, along with the private sector in the form of fee-for-service physicians, pharmacies, hospitals, clinics and diagnostic centres also have a critical role in providing health care to the citizens of Bangladesh. Currently there are around 3,000 registered private hospitals/clinics and about 5,100 registered diagnostic laboratories. In addition, around 50,000 traditional medicine practitioners, 90,000 homoeopathic practitioners, 170,000 drug shop attendants, and another 185,000 village doctors are providing health services to a large majority of the people of Bangladesh [4]. However, the lack of adequate regulations and oversight has undermined the full potential of the private sector and contributed to the usual provision of poor quality services[11].

The country’s health system is struggling to meet basic standards of care because of a shortage of skilled health workers, the disproportionate numbers of physicians and nurses, the large number of unregulated private service providers, and a large number of informal providers [4]. Further complicating the situation is the irregular supplies of drugs, inadequate public financing, high out of pocket expenses and an absence of proper monitoring and supervision mechanisms. Adding to Bangladesh’s already daunting challenge in achieving the goal of UHC is the increasing burden of non-communicable diseases, and the absence of any pre-payment risk pooling mechanisms. It is absolutely necessary that the country urgently address this critical situation through a comprehensive UHC agenda that ensures care for all citizens at an affordable price. Effective design and implementation of UHC is also conditional on an appropriate and well-defined M&E framework to measure and monitor the progress towards UHC.

This paper is part of a series of country case studies that aim to synthesize lessons learned from measuring and monitoring UHC in different contexts and to develop global guidance for the monitoring and measurement of UHC, including a set of core indicators and measurement approaches. This paper reviews the national health sector program’s result framework and provides an overview of the current status of Bangladesh in terms of UHC. The paper also makes specific recommendations for more effective tracking of the progress towards UHC.

**2. Universal health coverage: the policy context**

In recent years, there has been a general increase in media coverage about UHC and efforts to learn from neighboring countries, which has contributed to a policy-level dialogue on UHC. Despite this apparent momentum, there has been remarkably little implementation of any UHC initiative in the ground [11]. The first ever health financing strategy of the country was developed and approved in 2012 with a roadmap to achieve universal health coverage by 2032[12]. The strategy emphasizes an expansion of the coverage of prepayment schemes and increased public expenditure on health. Following the development of the strategy, the government is now planning for a subsidized health insurance scheme for about 31% of the population who are below the poverty line covering a range of services from outpatient consultations to common surgical interventions. Apart from this government-led initiative, several NGOs are piloting their own micro-health insurance schemes targeting low-income households. However the scale of these programmes and the products are not very encouraging. There is no evidence of any large-scale implementation with reasonable coverage and risk sharing. The benefit packages for most of the NGO schemes only cover basic curative services. The schemes neither focus on preventive care for emerging chronic diseases or expensive inpatient care, and in most cases the NGOs remain both insurer and provider[11].

**3. Monitoring and evaluation for UHC**

Results Framework for the HPNSDP and Source of data

The Health, Population and Nutrition Sector Development Programme (HPNSDP) 2011-2016 is the country’s third five-year health programme since it adopted the sector-wide approach (SWAp) in 1998. The country has established an annual process to assess progress of the sector program. The Planning Wing of the Ministry of Health and Family Welfare (MoHFW) is responsible for the annual program review based on a results framework (RF) with a set of well -defined and agreed upon indicators. Appropriate benchmarks and targets were set before the start of the programme[13]. A programme management and monitoring unit (PMMU) has also been established as a special unit to provide technical support to the Planning Wing[13]

In line with the overall priorities of HPNSDP, its result framework (Table S1 – end of file) mostly focused on indicators that measure access and utilization of essential health, population and nutrition services related to the health MDG for women and children. Our assessment of the results framework reveals that there is a gap in monitoring the critical health financing input although data to measure such indicators are readily available. At the output level, indicators measuring service access for non-communicable diseases and injuries are missing. At the outcome level, the present set of indicators suggests a strong preference for measuring intervention coverage provided at the primary level (e.g., rate of skilled birth attendance, ANC, PNC). There is no indicator related to the measurement of coverage of secondary or tertiary level health care services, or risk factors for non-communicable diseases. Most importantly, indicators that measure coverage of financial risk protection, a key UHC component, are totally absent. In terms of measuring equity, we found very few indicators at the outcome level having an equity dimension (geographical equity and income equity). At the goal level, there is no indicator that measures the health status of the general adult population or financial wellbeing of the households.

A majority of the indicators in the results framework, including almost all of the goal and outcome-level indicators, are measured with the data from periodic surveys carried by different government and non-government agencies. This huge dependence on surveys is because, the quality of the routine information has always been of questionable standard and also the routine system provides data from the public sector only. Moreover in the absence of a reliable civil registration system for measuring denominators of many standard indicators (births, deaths etc) programmes are forced to rely on surveys. Several programmes in the country use intercensal projections to estimate denominators for many indicators, e.g., for the immunization programme. However these projections may not be a very reliable estimate for the smaller units (district, sub-district) and thus do not help in regular monitoring.

So far, both the government and development partners have been very willing to invest in population-based surveys, even though they are generally expensive. A DHS has been conducted every 3-4 years since 1993. In the years between the DHSs, MoHFW also conducts Utilization of Essential Service Delivery (UESD) surveys, which largely follows DHS sampling procedure, albeit with a smaller sample, to provide comparable updates on coverage indicators[14]. The first Non-Communicable Disease Risk Factor Survey was carried out in 2010 [15]. There also have been two large surveys to measure maternal mortality, with the last one in 2010 covering 170,000 households[16]. UNICEF has also been doing its Multiple Indicators Coverage Surveys (MICS), with the latest in 2012-13[17]. Additional data is available from Urban Health Survey, Bangladesh Health Facility Surveys and EPI Coverage Evaluation Surveys[8, 9, 18, 19]. For monitoring expenditures on health, Bangladesh National Health Accounts[20] is currently the most common and reliable source of information. In addition, information is available from a number of research studies on healthcare expenditures and coping mechanisms as well as economic consequences[21-23]. The Household Income and Expenditure Survey (HIES) of Bangladesh, carried out once every five years by Bangladesh Bureau of Statistics (BBS), is the source of national data for OOPE[24].

M&E Framework for Universal Health Coverage:

In order to make Universal Health Coverage a reality it is very important that we monitor progress through a well-defined M&E framework. The indicators in the framework should address all levels of the health system performance and must be comparable with other countries while being adapted to the Bangladesh context. The current HPNSDP RF could be a basis for the UHC framework, which was developed through a vigorous national stakeholder consultation process and was cited as one of the best examples for monitoring results [26]. However as mentioned earlier the current RF does not address some of key aspects of UHC, hence we suggest including a few additional indicators (Table S1, end of file). A national stakeholder consultation process would be needed to reach agreement on a final set of indicators.

The country needs to essentially monitor key programme inputs and processes, as it will need to continue investing substantially in strengthening the capacity of its weak health system in order to make UHC a reality. Thus tracking health-financing inputs, density of health workforce, supplies of essential medicines, availability and functionality of key instruments, and use of the health information system would be critical. With introduction of prepayment schemes we expect service utilization to increase at all levels and it is also desirable and expected that the quality of service will improve in the context of increased competition to participate in the prepayment schemes. Thus it is important we measure utilization and quality of services provided at different levels of heath care facilities. We should continue measuring coverage of priority public-health interventions especially those for non-communicable diseases for all ages and gender. The service coverage indicators should be linked with the proposed service package to be covered by the national health insurance scheme, which, in turn, should be based on the country’s disease burden. It would also be necessary to measure effective coverage (i.e., affordable good quality care) rather than nominal coverage. The country will also need continuous feedback whether their efforts in achieving UHC are also contributing to progressive realization of the equity goals. Thus the framework must allow disaggregation of data. Another important area is to measure and monitor the financial risk protection coverage as an assessment of the direct effect of pre-payment financing mechanisms. At the impact level, it would be important to include indicators on chronic diseases and injury and on financial well-being[13].

**4. Progress towards UHC in Bangladesh**

We now describe the current situation of UHC in Bangladesh based on priority indicators from the suggested UHC framework. At the input level, health financing indicator data shows total healthcare expenditure of Bangladesh steadily increased from 1997 to 2007. This is natural as Bangladesh is experiencing strong economic growth in the last 10 years with GDP growth averaging 5.8% per annum [25]. The total health expenditure as a percentage of GDP grew from 2.7% in 1997 to 3.7% in 2011. The per capita health care expenditure in PPP-adjusted US$ increased from 20 in 1997 to 67 in 2011(Table S2). However, the growth of public health care financing is not encouraging. The public expenditure (actual) as a percentage of GDP remained at 1% throughout this period (Table S2) and currently constitutes only 26% of the total health care expenditures[20]. The growth in total health expenditures is thus mostly because of the accelerated growth in out of pocket expenditure (OOPE). In 1997, private health expenditure (more than 96% of which is borne by households’ out of pocket expenditure) constituted 57% of total health expenditure, which increased to 64% in 2007. In contrast, public expenditure declined from 36% of the total health care expenditure in 1997 to 26% in 2007[20]. An estimation from the latest Household Income and Expenditure Survey[24] of Bangladesh shows that out of pocket expenditure per capita is US$22.0 (unpublished), implying that 89% of the total health expenditure is directly made by households as out of pocket expenditure. During this period the share of total government budget in health decreased from 6.18% in 2009-10 to 4.26% in 2013-14. In sharp contrast there has been a significant increase in out-of-pocket expenditure, which however explains the increase in total health expenditure over the years (Figure S3). Such reliance on out of pocket expenditure poses a significant barrier to a large number of low-income people for accessing healthcare.

|  | 1997 | 2000 | 2003 | 2005 | 2007 | 2008 | 2009 | 2010 | 2011 |
| --- | --- | --- | --- | --- | --- | --- | --- | --- | --- |
| Total Health Expenditure in PPP$ per capita | 20 | 24 | 30 | 37 | 46 | 52 | 58 | 61 | 67 |
| Total Health Expenditure in US$ per capita | 9.2 | 10.1 | 11.5 | 13.7 | 16.2 | 19.4 | 22.4 | 24.8 | 26.5 |
| Total Health Expenditure as % of GDP | 2.7% | 2.8% | 3.0% | 3.2% | 3.4% | 3.5% | 3.7% | 3.7% | 3.7% |
| Mean annual growth rate in total health expenditure |  | 10% | 8% | 15% | 16% | - | - | - | - |
| Mean annual growth rate in GDP |  | 8% | 10% | 11% | 14% | - | - | - | - |
| Public expenditure on health as % of total health expenditure | 36% | 31% | 28% | 26% | 26% | 36% | 37% | 37% | 37% |
| Public expenditure on health as % of GDP | 1% | 1% | 1% | 1% | 1% | 1% | 1% | 1% | 1% |
| OOPP as % of total health expenditure | 57% | 59% | 61% | 64% | 64% | 62% | 61% | 61% | 61% |
| NGO expenditure as % of total health expenditure | 1% | 2% | 2% | 2% | 1% | - | - | - | - |
| External assistance to NGOs as % of total health expenditure | 5% | 7% | 9% | 8% | 8% | - | - | - | - |
| Other private expenditure as % of total health expenditure | 1% | 1% | 1% | 1% | 1% | - | - | - | - |

**Table S2**: Trends in health expenditure in Bangladesh, 1997-2011

Data Source: Bangladesh National Health Accounts 1997-2007, World Bank 2012

**Figure S3:** Trend in public expenditure and private expenditure in health as a percentage of total health expenditure in Bangladesh, 1997-2007

Data source: MoHFW, 2010

The density of doctors, nurses and midwives currently stands at 6 per 1000 population which is a fourth of the WHO recommended threshold level of 23 per 1000 population [23]. The country is also at the bottom of the list showing bed per 1000 population of different countries with 0.6 beds per 1000 population[26]. The 2011 health facility survey estimated that only 27% of hospitals had at least 75% of the essential medicines. About 7% of district hospitals (DH) and 16% of sub district hospitals (UHC) did not have any X-ray machines. Out of them 65% of x-rays in DHs and 52% of x-rays in UHCs are functional[27]

For several priority public-health interventions the country has reached fairly high levels of coverage with greater equity. For example, the country has achieved high vaccine coverage while reducing disparities significantly between different wealth quintiles. In 2011, 86% of children ages 12-23 months were immunized with all basic vaccines, which was 79% in the lowest wealth quintile and 94% in the highest wealth quintile (Figure S4 and S5). The primary treatment coverage for diarrhoea and acute respiratory infections (ARI) has also improved. In 2011 81% of under-five children with diarrhoea were treated with ORS. Among children with ARI, 35% were taken to a health facility or a health care provider and 71% received an antibiotic (Figure S6).

**Figure S4:** Percentage of children 12-23 months vaccinated within 12 months

Data source: BDHS 2011

**Figure S5:** Percentage of children 12-23 months vaccinated within 12 months by wealth quintiles

Data source: BDHS 2011

**Figure S6:** Percentage of children with illness who received care

Data source: BDHS 2011

Bangladesh’s progress with interventions to combat malnutrition has been mixed. According to the latest DHS, 90% of children are breastfed until the age two years and 64% of children less than age 6 months are exclusively breastfed. However, a mere 21% of children age 6-23 months are appropriately fed based on recommended infant and young child feeding practices[19]. The country has done less well with certain interventions that require relatively higher clinical care, For example, the rate of delivery assisted by skilled birth attendants is only 32%(Figure S7).

**Figure S7:** Skilled attendance at Delivery

Data source: BDHS 2004, 2007, 2011

Bangladesh has started to track the prevalence of major non-communicable diseases (NCD) as well as of risk factors that have the greatest impact on NCDs. BDHS 2011 survey reported that 32% of women and 19% of men older than 35 years of age are hypertensive. For diabetes these figures are 11.2 % and 10.7% respectively[19]. The country is also measuring effective coverage of interventions defined as the proportion of the population who needed a service that received it with sufficient quality to be effective [28] for certain chronic conditions. The same survey reported 20% of women with hypertension and 15% of women with diabetes had their conditions under control with medication (Figure S1). For men these figures are 16% and 10%[19].

**Figure S1:** Effective treatment coverage for Diabetes and Hypertension in women age 35 years and older

Data source: BDHS 2011

For risk factors, the Bangladesh NCD Risk Factor Survey 2010 reports that 75% of rural males 25 years and older used tobacco compared with 65% of urban males; for women, the proportions are 39% and 30%, respectively[15]. In terms of improved household financial wellbeing a study reported that in 2005 15.6% of households in Bangladesh faced catastrophic health expenditures[21, 22]. Every year catastrophic health expenses push around 3.8% of the population or 5.7 million people into impoverishment[23].

At the level of population impact, Bangladesh is well on its way to achieve the main targets of both MDG4 and 5, with plummeting rates of under-five and maternal mortality (Figure S2). Fertility is a third of what it was when Bangladesh gained its independence in 1971. However the rate of stunting is still high, though slowly declining. BDHS 2011 reported 41% of children under age 5 are stunted.

**Figure S2:** Trends in Under-5 Child Mortality

Data source: BDHS

**5. Conclusions and recommendations**

Universal Health Coverage (UHC) is considered by many as the next development goal for post 2015. Bangladesh faces enormous challenges in achieving UHC. It needs to reduce the huge gap between need and utilization, vastly improve the overall quality of health services and most importantly improve financial protection so that use of health services does not expose the user to financial hardship. It needs improvement both in access to effective preventive and curative interventions, particularly for the highest burden conditions and interventions most likely to result in financial hardship and impoverishment.

The country requires major reforms in the way health is currently financed. The Health Financing Strategy 2012-32[12] and Call for Action paper in the recently published Lancet Bangladesh series[6]both called for an increased budget allocation for health. The previous health-sector plan aimed to set the total health budget at 10% of the overall national budget, but that target was never realized. In reality, the proportion of the health budget as part of the national budget has been decreasing and stands at 4.9% in 2013-14 compared with 6.3% in 2009-10[12]. The Government needs to invest heavily in health to strengthen the capacity of its weak health system. It also urgently needs to introduce mandatory prepayment schemes for the formal sector employees and subsidized schemes for the poor as planned in the health financing strategy.

In terms of monitoring the progress of UHC, It is important that it identify achievable and measurable milestones adapted to its own context. The UHC M&E framework must be established through inclusive policy dialogue, and with support and alignment of the country and development partners and embedded in the national sector strategic plan. The framework should effectively reflect the country’s disease burden profile, health system capacity and level of economic development. Our review of the results framework suggests indicators currently being measured do not adequately span the range of preventive, curative and rehabilitative care needed for UHC. With the currently available information sources we are unable to measure the burden of many non-communicable conditions and the coverage of interventions provided at the secondary and tertiary level.

The Country needs to strengthen its information sources. Of the major information sources the routine health information system represents perhaps the most pressing area for improvement as it is likely to be the most crucial to the successful monitoring and evaluation of both the health sector programme and UHC. A key priority of the current health program is aimed at strengthening and improving the routine health information system (RHIS) so that information collected is complete and of good quality.

Recently, we have seen some consolidated efforts to strengthen RHIS, particularly with increasing use of technology for capturing, transmitting and processing data. The government has made substantial investments in expanding the infrastructure for RHIS. The Management Information System units of MoHFW are working with different national and international partners in strengthening the routine information system. A major modification has been made in the recording and reporting formats and also an electronic data capturing system has been introduced to more rapidly process information. However, efforts to build capacity of the workforce and to create an organizational culture that uses information for decision making has remained inadequate. There are also certain indicators such as those measuring risk behaviors that cannot be measured through RHIS. We will therefore, see a continued dependence on household and facility surveys for measurement of such indicators. Necessary steps need to be taken to strengthen the capacity of relevant government agencies so that they can conduct high quality surveys at regular frequencies. It is also important that we reconcile data from household surveys with other routine data sources to ensure the quality of the surveyed data. Strengthening civil vital registration system is almost as urgent as the need to strengthen the RHMIS. It is important that the country adopt legislation establishing continuous, permanent and compulsory civil registration systems.

Bangladesh also needs to strengthen the information sources for health financing data. Bangladesh National Health Accounts and Bangladesh Household Income and Expenditure Survey are being the two most important source of information for health financing. However the publication of Bangladesh National Health Accounts has been quite irregular. The last Bangladesh National health Accounts report was published in 2010 after a gap of 7 years. We need this information at regular interval. Bangladesh Household Income and Expenditure Survey should also look at the distribution of out-of-pocket expenditure across socioeconomic groups, in relation to care for specific diseases (diabetes, cancer, and cardiovascular disease.). Further analysis can be done to assess what share of poverty in Bangladesh can be explained by catastrophic health expenditures associated with out of pocket expenditure.

All current efforts for strengthening the routine health information sources are targeted towards public sector service delivery only and thus information on private sector services, which actually constitutes the bulk of the health services in the country, is mostly missing. It would be important to strengthen the MOHFW’s stewardship capacity so that information from the private sector is collected through routine health information system in the future. The country also needs to start monitoring the social and environmental determinants of health and sustainable development. There is an important role for a well-defined monitoring and evaluation framework to make UHC a reality. It is expected that the country will put the highest priority for effectively and reliably tracking the progress with UHC. A strong appreciation of what is needed to achieve UHC and political commitment from the highest levels in the country is an absolute prerequisite.

| **Box S1: Recommendations**   1. The country urgently needs to develop a monitoring framework for UHC that is based on the health sector’s result framework and embedded in the national health sector strategic plan. 2. The M&E framework for UHC must be established through inclusive policy dialogue, and with support and alignment of country and development partners. 3. The framework should effectively reflect country’s disease burden profile, health system capacity and level of economic development. 4. It should focus on measuring effective coverage rather than nominal coverage 5. All coverage indicators to be disaggregated by socioeconomic, demographic, region stratifiers. 6. National Health Accounts should be carried out regularly and must be institutionalized. 7. Must strengthen the MoHFW’s technical capacity stewardship role to oversee all surveys and ensure data from the private sector. 8. Country should intensify the current efforts in strengthening the RHIS and build an interoperable electronic health information system to be used in all level of health facilities. |
| --- |

**References**

1. World Health Organization. The World Health Report 2010 - Health systems financing: the path to universal coverage. Geneva.; 2010,. World Health Organization, .
2. UN. United Nations General Assembly, Sixty-seventh Session, 6 December 2012. Agenda item 123: Draft resolution on Global health and foreign policy: social protection and universal health coverage. 2012. .
3. Chowdhury, AM, Bhuiya A.M.,, Chowdhury ME, Rasheed S, Hussain Z, et al.,. (2013) The Bangladesh paradox: exceptional health achievement despite economic poverty. Lancet, 2013. 382(9906): p.: 1734-451745.
4. Ahmed, SM, Evans TG, Standing H, Mahmud S.M., et al., (2013) Harnessing pluralism for better health in Bangladesh. Lancet, 2013. 382(9906): p.: 1746-551755.
5. El Arifeen, S., et al.,, Christou A, Reichenbach L, Osman FA, Azad K, et al. (2013) Community-based approaches and partnerships: innovations in health-service delivery in Bangladesh. Lancet, 2013. 382(9909): p.: 2012-262026.
6. Adams, A.M., AM, Ahmed T, El Arifeen S, Evans TG, Huda T, et al.,. (2013) Innovation for universal health coverage in Bangladesh: a call to action. Lancet, 2013. 382(9910): p.: 2104-112111.
7. Adams, AM, Rabbani A, Ahmed S, Mahmood SS, Al-Sabir A.M.,, et al.,. (2013) Explaining equity gains in child survival in Bangladesh: scale, speed, and selectivity in health and development. Lancet, 2013. 382(9909): p.: 2027-372037.
8. NIPORT, Mitra and Associates, Macro International. Bangladesh Demographic and Health Survey 2007. Dhaka, Calverton: National Institute of Population Research and Training, Mitra and Associates, and Macro International, 2009.
9. NIPORT, Mitra and Associates, ORC Macro. Bangladesh Demographic and Health Survey 1999–2000. Dhaka, Calverton: National Institute of Population Research and Training, Mitra and Associates and ORC Macro, 2001.
10. NIPORT, Mitra and Associates, ORC Macro. Bangladesh Demographic and Health Survey 2004. Dhaka, Calverton: National Institute of Population Research and Training, Mitra and Associates, and Macro International, 2005.
11. Bangladesh Health Watch. Moving towards universal health coverage. Dhaka: James P Grant School of Public Health, and BRAC University, 2011.
12. MOHFW. Expanding social protection for health: towards universal coverage—health care fi nancing strategy 2012–2032. Dhaka: Health Economics Unit, Ministry of Health and Family Welfare, 2012.
13. Government of Bangladesh. Health, Population and Nutrition Sector Development Program (HPNSDP) July 2011 - June 2016: Program Implementation Plan (PIP), Vol I. Dhaka: Ministry of Health and Family Welfare, Bangladesh, 2011.
14. NIPORT, Utilization of Essential Service Delivery (UESD) Survey 2010. National Institute of Population Research and Training, Mitra and Associates, 2011. .
15. World Health Organization. Non-Communicable Disease Risk Factor Survey Bangladesh 2010. http://www.searo.who.int/bangladesh/publications/ncd_risk_factor_2010/en/ (accessed 20 January 2014).
16. National Institute of Population Research and Training (NIPORT), USAID, UN Population Fund, AusAID, Measure Evaluation, International Centre for Diarrhoeal Disease Research, Bangladesh (icddr,b). Bangladesh maternal mortality and health care survey 2010. Dhaka: NIPORT, Measure Evaluation, icddr,b, 2011.
17. BBS. 2009 Multiple Indicator Cluster Survey Bangladesh Bureau of Statistics, Dhaka, Bangladesh 2010 http://www.unicef.org/bangladesh/MICS-PP-09-v10.pdf (accessed 20 January 2014).
18. NIPORT, MEASURE Evaluation, icddr,b, ACPR. 2006 Bangladesh urban health surveyUrban Health Survey. Dhaka; Chapel Hill: National Institute of Population Research and Training, MEASURE Evaluation, International Centre for Diarrhoeal Disease Research, Bangladesh, Associates for Community and Population Research, 2008.
19. NIPORT, Mitra and Associates, ICF International. Bangladesh demographic and health survey 2011. Dhaka, Calverton: National Institute of Population Research and Training, Mitra and Associates, ICF International, 2013.
20. BNHA (2010). Bangladesh National Health Accounts, 1997-2007. Dhaka: Health Economics Unit, MOHFW, Government of Bangladesh.
21. van Doorslaer, E.,, O'Donnell O, Rannan-Eliya RP, Somanathan A, Adhikari SR, et al.,. (2007) Catastrophic payments for health care in Asia. Health Econ, 2007. 16(11): p.: 1159-841184.
22. van Doorslaer, E.,, O'Donnell O, Rannan-Eliya RP, Somanathan A, Adhikari SR, et al.,. (2006) Effect of payments for health care on poverty estimates in 11 countries in Asia: an analysis of household survey data. Lancet, 2006. 368(9544): p.: 1357-641364.
23. Xu, K.,, Evans DB, Kawabata K, Zeramdini R, Klavus J, et al.,. (2003) Household catastrophic health expenditure: a multicountry analysis. Lancet, 2003. 362(9378): p.: 111-7117.
24. BBS. 2011. Report of the Household Income and Expenditure Survey 2010. Bangladesh Bureau of Statistics, Dhaka.
25. The World Bank. GDP Growth (%) 2004-2012 Bangladesh. http://databank.worldbank.org/data . Accessed 15 Nov 2013.
26. World Bank. Hospital beds (per 1,000 people). http://data.worldbank.org/indicator/SH.MED.BEDS.ZS (accessed January 24, 2014).
27. 2011 Bangladesh Health Facility Survey. University of South Carolina, Associates for Community and Population Research, Dhaka, Bangladesh 2012 http://hpnconsortium.org/admin/essential/Bangladesh_Health_Facility_report_2011_Feb_12_V2.pdf (accessed 20 January 2014).

|  | | | |
| --- | --- | --- | --- |
| **Input and Processes** | **Output** | **Outcome** | **Impact** |
| **Health Financing**   1. Percent of MOHFW budget allocated to Upazilla level or below  - *Per capita total on health (PPP in int $)* - *Out of Pocket Expenditure as % of total expenditure on health* - *General government expenditure on health as a percentage of total expenditure on health* - *General government expenditure for health as a percentage of total government expenditure*   **Health workforce**   1. # of additional providers trained in midwifery at Upazila health facilities 2. Proportion of service provider positions functionally vacant at District, Upazila and below levels, by category  - *Density of nursing and midwifery personnel by districts (per 10 000 population)*   **Procurement and Supplies**   1. % of health facilities, by type, without stock-outs of essential medicines at a given point in time 2. % of facilities without stock-outs of contraceptives at a given point in time  - *Percent of facilities, by type, with 75% or more basic equipment functional*   **Infrastructure and Health Information**   1. Percent of health facilities (excluding CCs) having separate, improved toilets for female clients 2. MIS reports on service delivery published and disseminated annually  - *Psychiatric beds (per 10,000 population)* - *Density of radiotherapy units (per 1 000 000 population)* - *Percent of health facilities, by type reporting to national health information system* - *Civil registration coverage of births and deaths (%)* | **Service Access and readiness**   1. Number of Community Clinics with increasing number of service contacts over time 2. Number of comprehensive emergency obstetric care facilities with functional 24/7 services covering all districts 3. Percent of upgraded* union-level facilities able to provide basic EmOC services 4. Number of Upazillas with women targeted by improved voucher scheme for having institutional deliveries  - *Percent of facilities, by type, screening for a) hypertension and b) diabetes for adult clients*   **Service quality and safety**   1. Case fatality rate among admitted children 0-5 years with pneumonia who attended inpatient and emergency departments of IMCI facilities  - *Percent of deliveries ending in perinatal death* | **Health Service Coverage**   1. Percent of delivery by SBA 2. Antenatal care coverage (at least 4 visits) 3. Postnatal care within 48 hours of delivery by a medically trained provider 4. Contraceptive prevalence rate 5. Unmet need for Family planning 6. TB case detection rate 7. Proportion of births in health facilities by wealth quintiles 8. Use of modern contraceptives in low performing areas 9. Measles immunization coverage by 12 months 10. Percent of children (0-59 months) with pneumonia receiving antibiotics 11. Percent of children (6-59 months) receiving Vitamin A supplementation in the last 6 months   **Risk factors**   1. Rate of exclusive breastfeeding in infants up to 6 months 2. Percent of children 6-23 months fed with appropriate Infant and Young Child Feeding (IYCF) practices  - *Prevalence of raised blood pressure among adults aged>15 years* - *Prevalence of raised blood glucose among adults aged>15 years* - *Prevalence of current tobacco use among adults ages >15 years*   **Coverage of financial risk protection**   - *Percent of population under any prepayment scheme* | **Improved health status**   1. Infant mortality rate 2. Under 5 mortality rate 3. Neonatal mortality rate 4. Maternal mortality ratio 5. Total fertility rate 6. Prevalence of stunting among children <5yr 7. Prevalence of underweight among children <5yr 8. Prevalence of HIV in MARP   **Improved household financial wellbeing**   - *Percent of household facing impoverishment due to out of pocket expenditures on health*   **Other Indicators in the RF**   1. Percent of annual work plans with budgets submitted by Line directos by defined time period (July/Aug) 2. Performance report of Operational plan reviewed with policy makers, MOHFW, Directorates and Development partners, six monthly and annually 3. # of non-pool development partners submitting quarterly expenditure reports 4. % of project aid fund (e.g. development budget) disbursed annually and quarterly 5. % of Operational Plans with spending >80% of Annual Development Program allocation (annually) 6. % of serious audit objections settled within the last 12 months 7. Regulatory framework for accreditation of health facilities including hospitals (both in the public and private sectors) reviewed, updated and implemented 8. Number of Districts/Upazilla having functional Local Level Planning procedure |

**Note:** Additional indicators (presented in bullet points and italics) have been suggested to monitor the country’s progress towards universal health coverage

**Table S1:** Result framework of current health sector program with additional suggested indicators

Data Source: Government of Bangladesh. Strategic Plan for Health, Population and Nutrition Sector Development Program (HPNSDP) July 2011 - June 2016
